# Syndromic Surveillance and Bioterrorism-related Epidemics

**DOI:** 10.3201/eid0910.030231

**Published:** 2003-10

**Authors:** James W. Buehler, Ruth L. Berkelman, David M. Hartley, Clarence J. Peters

**Affiliations:** * Emory University Rollins School of Public Health, Atlanta, Georgia, USA; † University of Maryland School of Medicine, Baltimore, Maryland, USA; ‡ University of Texas Medical Branch, Galveston, Texas, USA

## Abstract

To facilitate rapid detection of a future bioterrorist attack, an increasing number of public health departments are investing in new surveillance systems that target the early manifestations of bioterrorism-related disease. Whether this approach is likely to detect an epidemic sooner than reporting by alert clinicians remains unknown. The detection of a bioterrorism-related epidemic will depend on population characteristics, availability and use of health services, the nature of an attack, epidemiologic features of individual diseases, surveillance methods, and the capacity of health departments to respond to alerts. Predicting how these factors will combine in a bioterrorism attack may be impossible. Nevertheless, understanding their likely effect on epidemic detection should help define the usefulness of syndromic surveillance and identify approaches to increasing the likelihood that clinicians recognize and report an epidemic.

Because of heightened concerns about the possibility of bioterrorist attacks, public health agencies are testing new methods of surveillance intended to detect the early manifestations of illness that may occur during a bioterrorism-related epidemic. Broadly labeled “syndromic surveillance,” these efforts encompass a spectrum of activities that include monitoring illness syndromes or events, such as medication purchases, that reflect the prodromes of bioterrorism-related diseases (*1*–*9*). The Centers for Disease Control and Prevention (CDC) estimates that, as of May 2003, health departments in the United States have initiated syndromic surveillance systems in approximately 100 sites throughout the country (T. Treadwell, CDC, pers. comm.). The goal of these systems is to enable earlier detection of epidemics and a more timely public health response, hours or days before disease clusters are recognized clinically, or before specific diagnoses are made and reported to public health authorities. Whether this goal is achievable remains unproved (*4*,*5*,*10*).

Establishing a diagnosis is critical to the public health response to a bioterrorism-related epidemic, since the diagnosis will guide the use of vaccinations, medications, and other interventions. Absent a bioterrorism attack, predicting whether syndromic surveillance will trigger an investigation that yields a diagnosis before clinicians make and report a diagnosis is not possible. Our objective is to consider the mix of hypothetical factors that may affect the detection of epidemics attributable to CDC category A bioterrorism agents (*11*).

## Establishing a Diagnosis

**Figure 1:**
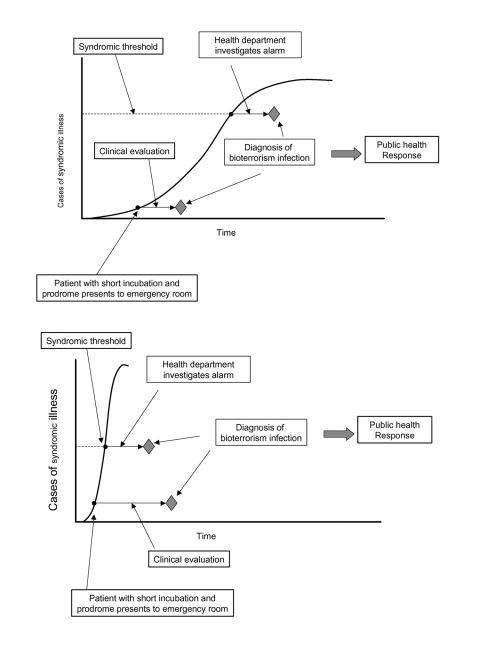
Number of cases of syndromic illness by time in a hypothetical bioterrorism attack and two pathways to establishing a diagnosis: syndromic surveillance coupled with public health investigation (upper pathway) and clinical and diagnostic evaluation of patients with short-incubation period disease (lower pathway). A, scenario favoring earlier detection by means of clinical evaluation. B, scenario favoring earlier detection by means of syndromic surveillance.

Two pathways to establishing a diagnosis are described by the scenarios below and in Figure 1, using a single, clandestine dissemination of an anthrax aerosol as an example:

## Detection through Syndromic Surveillance

The early signs of inhalational anthrax include nonspecific symptoms that may persist for several days before the onset of more severe disease (*12*). Patients with prodromal illnesses seek outpatient care and are assigned nonspecific diagnoses such as “viral syndrome.” Data on patients fitting various syndromic criteria are transferred to the health department and tested for aberrant trends. This process “flags” that a statistical detection threshold has been exceeded. Epidemiologists conclude that a preliminary investigation is warranted and collect blood for culture from several patients. Within 18 hours, one culture yields a presumptive diagnosis of anthrax, prompting a full-scale response.

## Detection through Clinician Reporting

Some persons in whom inhalational anthrax develops will have short incubation periods and prodromes (*12*). Respiratory distress occurs in one such person, and he is hospitalized. Routine admission procedures include blood cultures. Within 18 hours, a presumptive diagnosis of anthrax is made. The patient’s physician informs the local health department, prompting a full-scale response.

**Figure 2:**
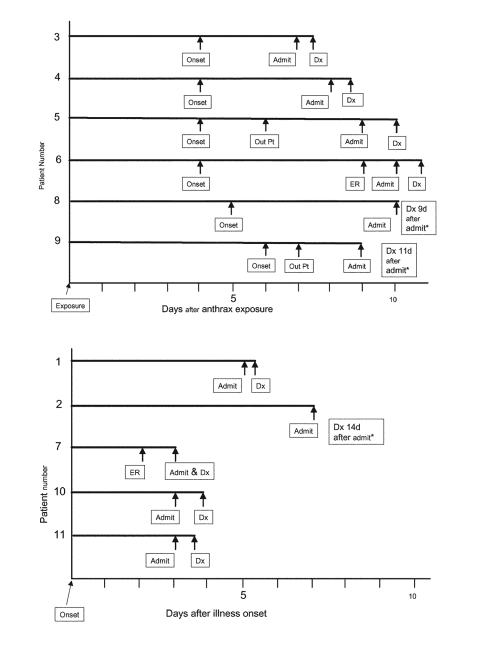
Timeline to presumptive anthrax diagnosis, 11 patients with inhalational anthrax, 2001, United States. Abbreviations: Dx, diagnosis; OutPt, outpatient visit followed by discharge home; ER, emergency room visit followed by discharge home. *Diagnosis delayed—initial blood cultures were negative in three patients who received antibiotic therapy before culture specimens were collected, requiring use of special diagnostic tests. For patients 1–10, case numbers correspond to those in report by Jernigan et al. (*13*); patient 11 reported by Barakat et al. (*14*). A, timeline begins with presumed date of anthrax exposure, available for six patients. B, timeline begins with day of illness onset for five patients without recognized date of exposure.

In practice, how a bioterrorism attack might be detected and diagnosed will probably be more complex. Published descriptions of 11 persons with inhalational anthrax in the United States in 2001 (*13*–*19*) provide some insight into this issue (Table 1 and Figure 2),^1^ even though that epidemic was too small and geographically diffuse to be detectable by syndromic surveillance. For six patients with known dates of exposure, the median duration between exposure and symptom onset was 4 days (range 4–6 days). The median duration between onset and the initial healthcare visit was 3 days (*20*) (range 1–7 days), and the median duration between onset of symptoms and hospitalization was 4 days (range 3–7 days). Two of the 11 patients visited emergency departments and were sent home with diagnoses of gastroenteritis or viral syndrome 1 day before admission. In one patient, a blood culture obtained in the emergency room was read as positive for gram-positive bacilli the following day, which prompted recall of the patient. The culture was subsequently confirmed as positive for *Bacillus anthracis.* Two other patients were seen by primary care physicians and sent home with diagnoses of viral syndrome or bronchitis 2–3 days before admission, including one patient who was begun on empiric antibiotic therapy. For seven other patients, initial emergency room or hospital visits led directly to admission. In addition to the patient whose blood culture was obtained in an emergency room, seven others had not received prior antibiotic therapy, and *B. anthracis* was presumptively identified from blood within 24 hours of culture. One of these seven patients was the index patient, in whom *B. anthracis* was also recognized in cerebrospinal fluid within 7 hours of specimen collection. Three other patients had received antibiotics before blood cultures were taken (one as an outpatient and two at the time of hospital admission), requiring alternative diagnostic methods.

**Table 1:** Outcome of initial contact with health care for anthrax-related illness and timing of anthrax diagnosis, 11 patients with inhalational anthrax, 2001^a^

Disposition after initial medical care	No. of patients
Admitted to hospital	7
Discharged home from ER, subsequent hospital admission	2
Discharged home from outpatient provider, subsequent hospital admission	2
Total	11
	
Anthrax diagnosis	
Blood or CSF culture on hospital admission, presumptive diagnosis <24 h	7
Blood culture from preceding ER visit, patient recalled for admission	1
Prior antibiotic therapy; clinical suspicion of anthrax; specialized test required to establish diagnosis	3
Total	11

^a^ER, emergency room; CSF, cerebrospinal fluid.

Despite the small number of patients, their experience offers four lessons for detecting an epidemic of inhalational anthrax. First, a key objective of syndromic surveillance is to detect early-stage disease, but fewer than half of these patients sought care before hospitalization was necessary, and the interval between such care and admission was relatively narrow (1–3 days). This finding suggests that syndromic surveillance data must be processed, analyzed, and acted upon quickly if such data are to provide a clue to diagnosis in advance of late-stage disease. Second, emergency room data are a common source for syndromic surveillance, but detecting an increase in visits coincident with hospital admission may not provide an early warning because the time needed to process surveillance data and investigate suspected cases would be at least as long as the time for admission blood cultures to be positive for *B. anthracis.* Blood cultures are likely to be routine for patients admitted with fever and severe respiratory illness, regardless of whether anthrax is considered as a diagnostic possibility, and *B. anthracis* grows readily in culture in the absence of prior antibiotic therapy, as observed in most of these patients. Thus, if emergency room data are to be useful in early detection of an anthrax epidemic, those data would need to be for visits that occur before hospital care is required—a pattern observed in only two patients. Third, the four patients who received early care and were discharged to their homes were assigned three different diagnoses, which suggests that syndromic surveillance systems must address the potential variability in how patients with the same infection may be diagnosed during the prodrome phase. Fourth, rapid diagnosis after hospitalization was possible only in those patients who had not received antibiotics before cultures were taken. This finding emphasizes the importance of judicious use of antibiotics in patients with nonspecific illness.

In addition to the specific attributes of individual bioterrorism agents, multiple considerations will shape the recognition of a bioterrorism-related epidemic. Five of these attributes follow.

### Size

Syndromic surveillance would not detect outbreaks too small to trigger statistical alarms. Size would be affected by the virulence of the agent, its potential for person-to-person transmission, the extent and mode of agent dissemination, whether dissemination occurs in more than one time or place, and population vulnerability.

## Population Dispersion

How persons change locations after an exposure will affect whether disease occurs in a concentrated or wide area, and thus whether clustering is apparent to clinicians or detectable through syndromic surveillance at specific sites.

### Health Care

The more knowledgeable providers are about bioterrorism agents, the greater the likelihood of recognition. Routine diagnostic practices or access to reference laboratories may affect the timeliness of diagnosis for some diseases. Familiarity with reporting procedures would increase prompt reporting of suspected or diagnosed cases.

### Syndromic Surveillance

Syndromic surveillance will be affected by the selection of data sources, timeliness of information management, definition of syndrome categories, selection of statistical detection thresholds, availability of resources for followup, recent experience with false alarms, and criteria for initiating investigations.

### Season

A fifth key attribute is seasonality. An increase in illness associated with a bioterrorism attack may be more difficult to detect if it occurs during a seasonal upswing in naturally occurring disease.

Agent- and disease-specific attributes may be among the most important factors affecting detection and diagnosis (Table 2). The incubation period and its distribution in the population will affect the rate at which new cases develop (*21*) and thus how quickly an alarm threshold is exceeded or whether clinicians recognize a temporal and geographic cluster. If a disease has a short prodrome, the chance is increased that a patient would be hospitalized and a definitive evaluation initiated before an increase in cases triggered a surveillance alarm. Alternatively, if a disease has a relatively long prodrome, chances are greater that prediagnostic events (e.g., purchase of medications or use of outpatient care for nonspecific complaints) would accrue to levels that exceed syndromic surveillance thresholds, before definitive diagnostic evaluations are completed among patients with more severe disease. Arousing clinical suspicion for a particular diagnosis will depend on the specificity of both the early and late stages of illness as well as the presence or absence of a typical feature that should alert clinicians to the diagnosis, such as mediastinal widening in inhalational anthrax (12). If a routinely performed test is apt to be diagnostic in a short time (e.g., the blood culture in anthrax), a rapid diagnosis is likely, even in the absence of clinical suspicion. If routine tests are unlikely to yield a rapid diagnosis (e.g., the blood culture for the cause of tularemia, *Francisella tularensis* [22]), or if the diagnosis requires a special test (e.g., the hemorrhagic fever viruses [23]), a diagnosis may be delayed if not immediately considered.

**Table 2:** Characteristics of bioterrorism-related epidemics that affect detection through clinical recognition versus syndromic surveillance

Characteristics^a^	Clinical recognition^b^	Syndromic surveillance^c^
Duration and variability of incubation period	Broader distribution of incubation period increases likelihood that patient with short incubation-period disease would be diagnosed before a statistical threshold of syndromic cases is exceeded.	More narrow distribution of incubation period—leading to a steeper epidemic curve in the initial phase—increases likelihood that statistical threshold would be exceeded sooner.
Duration of nonspecific prodromal phase	Shorter prodrome increases likelihood of recognition or diagnosis at more severe or fulminant stage.	Longer prodrome increases likelihood that increase in syndromic manifestations would be detectable and that recognition of more severe stage (at which a diagnosis is more apt to be made) would be delayed.
Presence or absence of clinical sign that would heighten suspicion of diagnosis	Presence increases likelihood of earlier clinical recognition and diagnosis (e.g., mediastinal widening on chest x-ray in inhalational anthrax).	Absence decreases likelihood that diagnosis would be considered clinically, increasing opportunity for earlier detection by means of syndromic surveillance.
Likelihood of making diagnosis in the course of routine evaluation	If diagnosis is apt to be made in the course of a routine diagnostic evaluation (not dependent on clinical suspicion of specific bioterrorism infection), early diagnosis through clinical care is likely.	If diagnosis is dependent on the use of a special test that is unlikely to be ordered in the absence of clinical suspicion of diagnosis, then diagnosis in clinical care may be delayed, increasing the opportunity for early detection through syndromic surveillance.

^a^Infection or disease attributes that may affect detection of an epidemic. ^b^Increases likelihood of initial detection through routine clinical care and reporting. ^c^Increases likelihood of initial detection through syndromic surveillance.

The public health benefit resulting from early detection of an epidemic is likely to vary by disease. If a disease has a relatively wide distribution of potential onsets, early recognition provides greater opportunity to administer prophylaxis to exposed persons. For example, based on data from the Sverdlovsk incident (*24*), Brookmeyer and Blades estimated that use of antibiotic prophylaxis during the 2001 anthrax outbreak prevented nine cases of inhalational disease among exposed persons (*25*). If the incubation period of a disease has a relatively narrow distribution, early recognition may offer little opportunity for postexposure prophylaxis, although a potential benefit would remain for alerting healthcare providers and informing their care of others with similar symptoms. This pattern of illness is apt to result from exposure to an *F. tularensis* aerosol, which would likely result in an explosive epidemic with an abrupt onset and limited duration (*22*).

## Detecting Specific Bioterrorism Epidemics and Agents

The attributes of the CDC category A bioterrorism agents that affect their detection, as well as the benefits of early detection, are summarized below, on the basis of potential bioterrorism-related epidemic profiles developed by experts (*12*,*22*,*23*,*26*–*28*). These profiles reflect current knowledge of these diseases; their epidemiology might differ if novel modes of dissemination or preparation were employed. Each disease has attributes that could increase or decrease the likelihood of early outbreak recognition through either clinical diagnosis or syndromic surveillance.

### Inhalational Anthrax

The distribution of the incubation period for inhalational anthrax can be relatively broad as observed in Sverdlovsk (2–43 days); most cases occur within 1–2 weeks after exposure (*24*). In the 2001 U.S. outbreak, the distribution of incubation periods was more limited, 4–6 days, although later-onset cases may have been averted by antibiotic prophylaxis (*25*). The nonspecific prodrome for anthrax may last from several hours to several days. Taken together, these data suggest that the initial slope of an epidemic curve may be comparatively gradual during the first week, leading to slower recognition through syndromic surveillance than for other infections caused by bioterrorist agents with pulmonary manifestations, such as tularemia or pneumonic plague (*22*,*28*). In contrast, mediastinal widening on chest x-ray or computed tomographic scan or Gram stain of cerebrospinal or pleural fluid should lead an alert and knowledgeable physician to consider the diagnosis of anthrax, even though these tests may not be conducted until relatively late in the clinical course. *B. anthracis* is likely to be detected quickly in cultures, favoring clinical recognition. Retrospective analysis of data from 2001 showed that inhalational anthrax can be distinguished from influenzalike illness or community-acquired pneumonia by using an algorithm that combines clinical and laboratory findings (*20*), although the practical utility of this approach is untested. In addition to permitting antibiotic use among ill persons, early recognition would enable postexposure antibiotic prophylaxis (*12*,*25*).

### Tularemia

The typical incubation period for tularemia is relatively narrow after a person is exposed to aerosolized *F***.** *tularensis,* with abrupt onset of nonspecific febrile illness, with or without respiratory symptoms, in 3–5 days (range 1–14 days), followed by rapid progression to life-threatening pneumonitis (*22*). This relatively narrow incubation period for most patients and rapid progression to severe disease would lead to a rapid increase in cases after a large and acute exposure. Finding a number of such cases in a short interval should trigger both syndromic surveillance alarms and clinical suspicion. *F. tularensis* is a slow-growing and fastidious organism and may take up to 5 days after inoculation to be detectable, if it is detected at all, in a routinely processed blood culture. The use of special laboratory techniques may be required, delaying the likelihood of detection in the absence of clinical suspicion. After an epidemic is recognized, specific antibiotic therapy is recommended for exposed persons in whom a febrile illness develops (*22*).

### Pneumonic Plague

Exposure to aerosolized *Yersinia pestis* results in pneumonic plague, which has a typical incubation period of 2 to 4 days (range 1–6 days). The disease has a relatively short prodrome, followed by rapidly progressive pneumonia (*28*), which would lead to a rapid increase in cases at the onset of an epidemic. Standard clinical laboratory findings are nonspecific, which alone might not prompt clinical suspicion, but microscopic examination of a sputum smear may show characteristic findings, which should prompt consideration of the diagnosis. Cultures of blood or sputum are apt to show growth within 24 to 48 hours, but routine procedures may misidentify *Y. pestis* unless the diagnosis is suspected and special attention is given to specimen processing. Confirming the diagnosis depends on special tests available through reference laboratories. Treatment the first day of symptoms is generally considered necessary to prevent death in pneumonic plague, so early recognition of an aerosol plague attack would enable life-saving use of antibiotics in febrile patients and prophylaxis of contacts (*28*).

### Botulism

Foodborne botulism typically has a relatively narrow incubation period (12–72 hours), which may vary from 2 hours to 8 days, depending on the inoculum. For the three known cases of inhalational botulism attributed to a relatively low exposure to aerosolized toxin, the incubation period was approximately 72 hours (*26*). The characteristic clinical picture of descending paralysis should prompt consideration of botulism, and this unique pattern among bioterrorism agents lends itself to a specific syndrome category. However, the illness may be misdiagnosed, as observed in a large foodborne outbreak of botulism in 1985; 28 persons who had eaten at a particular restaurant and in whom botulism had developed were assigned other diagnoses before the geographically dispersed outbreak was recognized and publicized in the media (*26*,*29*). Symptoms of inhalational botulism, with choking, dysphagia, and dysarthria dominating the clinical picture, may differ from those associated with ingestion of toxin and complicate recognition of the disease. Specialized testing for botulinum toxin is available at a limited number of state laboratories and CDC. Postexposure prophylaxis is limited by the scarcity of, and potential for, allergic reactions to botulinum antitoxin, leading to recommendations that exposed persons be observed carefully for early signs of botulism, which should prompt antitoxin use (*26*). Antitoxin should be given as early as possible, another fact that highlights the importance of early detection. Depending on the level of exposure and the geographic dispersion of affected persons, syndromic surveillance for characteristic neurologic symptoms could aid outbreak detection, or the occurrence of an epidemic might be obvious to clinicians.

### Smallpox

The incubation period of smallpox is usually 12–14 days but may range from 7 to 17 days. The early symptomatic phase includes a severe febrile illness and appearance of a nonspecific macular rash over a 2- to 4-day period, followed by evolution to a vesicular and then pustular rash over the next 4 to 5 days (*27*). Thus, the initial phase of smallpox may lend itself to detection through surveillance of a febrile rash illness syndrome. Once smallpox is suspected, the virus can be rapidly detected by electron microscopic examination of vesicular or pustular fluid, if laboratory resources for electron microscopy are available, or by polymerase chain reaction, if the necessary primers are available. Contacts can be protected by vaccination up to 4 days after exposure. Discourse is substantial about the relative merits of pre-event versus postevent vaccination (*27*,*30*–*33*). Syndromic surveillance may show an increase in febrile rash illness, although once the characteristic rash appears, the diagnosis should be quickly established.

## Viral Hemorrhagic Fevers

This category includes multiple infectious agents that range from having a relatively broad to narrow incubation period (e.g., Ebola, 2–21 days; yellow fever 3–6 days). These diseases present with nonspecific prodromes that may have an insidious or abrupt onset. In severe cases, the prodrome is followed by hypotension, shock, central nervous system dysfunction, and a bleeding diathesis. The differential diagnosis includes a variety of viral and bacterial diseases. Establishing the diagnosis depends on clinical suspicion and the results of specific tests that must be requested from CDC or the U. S. Army Medical Research Institute of Infectious Diseases. The value of postexposure prophylaxis with antiviral medications is uncertain, and (with the exception of yellow fever, for which a vaccine is available) response measures are limited to isolation and observation of exposed persons, treatment with ribavarin (if the virus is one that responds to that antiviral drug), and careful attention to infection control measures (*23*). Patients seen with symptoms during the prodromal phase may not clearly fit into a single syndrome category, but syndromic surveillance focused on the early signs of a febrile bleeding disorder would be more specific.

One of the biggest concerns about syndromic surveillance is its potentially low specificity, resulting in use of resources to investigate false alarms (*6*,*10*). Specificity for distinguishing bioterrorism-related epidemics from more ordinary illness may be low because the early symptoms of bioterrorism-related illness overlap with those of many common infections. Specificity for distinguishing any type of outbreak from random variations in illness trends may be low if statistical detection thresholds are reduced to enhance sensitivity and timeliness. The likelihood that a given alarm represents a bioterrorism event will be low, assuming that probability of such an event is low in a given locality. Approaches used to increase specificity include requiring that aberrant trends be sustained for at least 2 days or that aberrant trends be detected in multiple systems (*2*). Another approach to enhancing specificity would be to focus surveillance on the severe phases of disease, since the category A bioterrorism infections are more likely than many common infections to progress to life-threatening illness. For those diseases that are likely to progress rapidly, such as pneumonic plague, syndromic detection of severe disease (e.g., through emergency room visits, hospital admissions, or deaths) may be more feasible than detection aimed at early indicators before care is sought (e.g., purchases of over-the-counter medications) or when illness is less severe (e.g., primary care visits). Whether detection of syndromic late-stage disease offers an advantage over detection through clinical evaluation will depend on the attributes of the infections and diagnostic resources, as described above.

Predicting how the mix of relevant factors would combine in a given situation to affect the recognition of a bioterrorism-related epidemic is difficult, although mathematical models may provide further insight (*5*). The most important factors affecting early detection are likely to be the rate of accrual of new cases at the outset of an epidemic, geographic clustering, the selection of syndromic surveillance methods, and the likelihood of making a diagnosis quickly in clinical practice.

Ongoing efforts to strengthen the public health infrastructure (*34*,*35*) and to educate healthcare providers about bioterrorism diseases and reporting procedures should strengthen the ability to recognize bioterrorism outbreaks. For example, in New Jersey in 2001, reporting of two early cases of cutaneous anthrax was delayed until publicity about other anthrax cases prompted physicians to consider the diagnosis and notify the health department, suggesting that opportunities for earlier use of postexposure prophylaxis were missed (*36*). In addition, while the importance of new diagnostic tools, including rapid tests, should be emphasized (*37*), the essential role of existing diagnostic techniques should not be overlooked. Clinical suspicion is critical, and a key prompt for arousing clinical suspicion may be the microscopic examination of a routinely collected specimen, as occurred in the index case of the 2001 anthrax outbreak, when a Gram stain of the cerebrospinal fluid led to the diagnosis (*15*). However, as recently highlighted by the Institute of Medicine, the use of basic diagnostic tests has decreased because of efforts to reduce the costs of care, the increasing use of empiric broad-spectrum antibiotic therapy, and federal laboratory regulations, such as the Clinical Laboratory Improvement Amendments of 1988, which have discouraged laboratory evaluation in some clinical settings (*38*).

While we have focused on the role of syndromic surveillance in detecting a bioterrorism-related epidemic, other uses of syndromic surveillance include detecting naturally occurring epidemics, providing reassurance that epidemics are not occurring when threats or rumors arise, and tracking bioterrorism-related epidemics regardless of the mode of detection (*4*,*6*,*10*). Syndromic surveillance is intended to enhance, rather than replace, traditional approaches to epidemic detection. Evaluation of syndromic surveillance to consider the spectrum of potential uses is essential. A certain level of false alarms, as the result of either syndromic surveillance or calls from clinicians, will be necessary to ensure that opportunities for detection are not missed. Efforts to enhance the predictive value of syndromic surveillance will be offset by costs in timeliness and sensitivity, and defining the right balance in practice, particularly in the absence of an accurate assessment of bioterrorism risk, will be essential.

Two committees of the National Academies have recommended more careful evaluation of the usefulness of syndromic surveillance before it is more widely implemented (*5*,*38*). Because the epidemiologic characteristics of different bioterrorism agents may vary in ways that affect the detection of epidemics, these evaluations should address the epidemiology of specific bioterrorism agents. Efforts to detect bioterrorism epidemics at an early stage should not only address the development of innovative new surveillance mechanisms but also strengthen resources for diagnosis and enhance relationships between clinicians and public health agencies—relationships that will ensure that clinicians notify public health authorities if they suspect or diagnose a possible bioterrorism-related disease.

## Footnotes

1 For interval calculations, if reported event dates were discrepant in different case reports, dates reported by Jernigan et al. (*13*) were used.
